# Species differences in brain gene expression profiles associated with adult behavioral maturation in honey bees

**DOI:** 10.1186/1471-2164-8-202

**Published:** 2007-06-29

**Authors:** Moushumi Sen Sarma, Charles W Whitfield, Gene E Robinson

**Affiliations:** 1 Neuroscience Program, Institute for Genomic Biology, Department of Entomology, University of Illinois, 505 S. Goodwin Avenue, Urbana, Illinois 61801, USA

## Abstract

**Background:**

Honey bees are known for several striking social behaviors, including a complex pattern of behavioral maturation that gives rise to an age-related colony division of labor and a symbolic dance language, by which successful foragers communicate the location of attractive food sources to their nestmates. Our understanding of honey bees is mostly based on studies of the Western honey bee, *Apis mellifera*, even though there are 9–10 other members of genus *Apis*, showing interesting variations in social behavior relative to *A. mellifera*. To facilitate future in-depth genomic and molecular level comparisons of behavior across the genus, we performed a microarray analysis of brain gene expression for *A. mellifera *and three key species found in Asia, *A. cerana, A. florea *and *A. dorsata*.

**Results:**

For each species we compared brain gene expression patterns between foragers and adult one-day-old bees on an *A. mellifera *cDNA microarray and calculated within-species gene expression ratios to facilitate cross-species analysis. The number of cDNA spots showing hybridization fluorescence intensities above the experimental threshold was reduced by an average of 16% in the Asian species compared to *A. mellifera*, but an average of 71% of genes on the microarray were available for analysis. Brain gene expression profiles between foragers and one-day-olds showed differences that are consistent with a previous study on *A. mellifera *and were comparable across species. Although 1772 genes showed significant differences in expression between foragers and one-day-olds, only 218 genes showed differences in forager/one-day-old expression between species (p < 0.001). Principal Components Analysis revealed dominant patterns of expression that clearly distinguished between the four species but did not reflect known differences in behavior and ecology. There were species differences in brain expression profiles for functionally related groups of genes.

**Conclusion:**

We conclude that the *A. mellifera *cDNA microarray can be used effectively for cross-species comparisons within the genus. Our results indicate that there is a widespread conservation of the molecular processes in the honey bee brain underlying behavioral maturation. Species differences in brain expression profiles for functionally related groups of genes provide possible clues to the basis of behavioral variation in the genus.

## Background

The western honey bee, *Apis mellifera*, is a highly social insect that has emerged as one of the model organisms for using genomics to study the mechanisms and evolution of social behavior [1]. Honey bees are known for several striking social behaviors [2,3]. For example, adult worker honey bees display a complex pattern of behavioral maturation that involves working in the hive when young and foraging when older; this gives rise to an age-related division of labor in honey bee colonies that is thought to be one of the factors contributing to the spectacular ecological success of social insects [4]. Honey bees also are known for their symbolic dance language, by which successful foragers communicate the location of attractive food sources to their nestmates [3,5]. Behavioral maturation in adult honey bees is associated with coordinated changes in expression of thousands of genes in the brain [6-8], and a few genes have been identified that have causal effects [9-11]. Molecular or neural components of honey bee dance language have not yet been identified.

Our understanding of honey bees is mostly based on studies of *A. mellifera*, even though there are as many as 9–10 other members of genus *Apis *[12-14]. *Apis *is an ancient lineage of bees that evolved in tropical Eurasia ca. 8–11 million years ago [12] some migrated north and west, reaching Europe by the end of the Pleistocene epoch, 10,000 yr ago [15]. Phylogenetic analyses partition the genus into three groups. The cavity nesters form a monophyletic group and consequently *A. mellifera *and *A. cerana *are more closely related to one another than to *dorsata *and *florea *[12-14]. *A. dorsata *and *A. florea*, two open-nesting species, are in separate lineages, with *florea *part of the most basal lineage in the genus [14].

**Figure 1:**
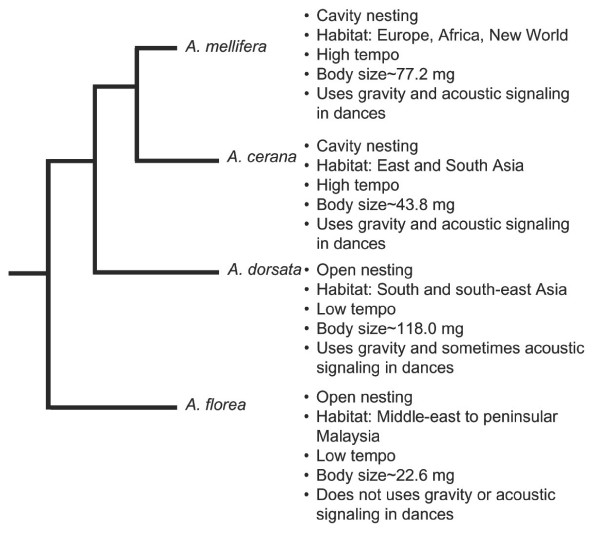
Known differences in ecology, behavior and physiology among the four species of honey bees studied: *A. mellifera, A. cerana, A. florea *and *A. dorsata*. Worker "tempo" is measured in terms of colony biomass, daily energy consumption, metabolic rate in terms of mass loss of mass loss [16]. Habitat and nesting data [13], body mass [16], dance language data [37–40].

Other *Apis *species display a rich combination of similarities and differences in behavior and ecology relative to *A. mellifera *[13]. Studies are limited, but there is some evidence that workers in other *Apis *species also show patterns of behavioral maturation similar to *A. mellifera *[3]; in all species studied, young workers perform nest work and older workers forage [16]. Conversely, comparative studies of key members of the genus, *A. mellifera, A. cerana, A. florea *and *A. dorsata*, have revealed marked differences in dance language, habitat, nesting habit, body size and worker "tempo" (Figure 1).

To facilitate future comparative studies of honey bee behavior at the genomic and molecular level, we performed a microarray analysis comparing brain gene expression of foragers with that of newly eclosed one-day-old adult bees ("one-day-olds") for *A. mellifera, A. cerana, A. florea *and *A. dorsata*. A previous study demonstrates that of the different age groups studied for *A. mellifera*, one-day-olds form perhaps the most discrete and cohesive group in terms of brain gene expression profiles [8]. In addition, a significant amount of brain maturation occurs between pupation and 4 weeks (foraging age) as evidenced by neuropil expansion and lengthening and branching of dendrites of Kenyon cells of mushroom bodies [17]. Also among adult groups compared, the most consistent differences in all parameters of brain maturation were seen between one-day-olds and foragers.

Genomic resources are currently very limited to a tiny fraction of the animal species available for study. Only one species of honey bee, *A. mellifera*, has a custom-designed microarray, made from an *A. mellifera *brain EST library [18] and representing ~ 5500 genes or ~ 50% of the currently annotated genes in the *A. mellifera *genome [15]. We show that this *A. mellifera *cDNA microarray can be used effectively for cross-species comparison of behavioral maturation within the genus. Brain gene expression profiles between foragers and one-day-olds showed differences that are consistent with a previous study on *A. mellifera *[8] and were comparable across species. Species differences in brain expression profiles for functionally related groups of genes provide clues to the basis of behavioral variation in the genus.

## Results and discussion

### Species differences in hybridization efficiency

A total of 4432 genes that represented 63% of the genes present in the array passed through our stringent analysis filters. This suggested the suitability of the array for interspecies comparisons. Only those spots which hybridized above the mean threshold intensity for all species were used (see Methods). The number of cDNA spots (genes) hybridizing above threshold fluorescence intensity was less for the three Asian species compared to *A. mellifera *(*A. mellifera *5595, *A. cerana *5202, *A. florea *4556, *A. dorsata *4535). Performances of the four species on the microarrays were comparable and in keeping with known differences and estimated approximate evolutionary distance. Thus *A. cerana*, sharing a monophyletic origin with *A. mellifera *[12-14], apparently displayed higher hybridization efficiency compared to the more distantly related *A. florea *and *A. dorsata*.

### Similarities in age-dependent differences in brain gene expression

We compared brain gene expression between foragers and one-day-olds of each colony, within each species. That is, the two samples hybridized on each array always belonged to the same species. This was done to avoid "false positives" that could arise due to interspecific differences in hybridization efficiency to the microarray [19,20]. We calculated gene expression ratios of forager to one-day-old brain expression levels for every gene and each replicate pair of arrays (data not shown; mean ratios available in an online repository [21]). Any difference detected between species using this ratio cannot be attributed specifically to either foragers or one-day-olds.

To measure replicability across experiments, we compared our data with a previous study [8]. Of the genes found to be significantly regulated between foragers and one-day-olds in each of the species in the current study, 58 – 75% also had been found to be significantly regulated between foragers and one-day-olds in reference 8 (Table 1). The higher number of genes significantly regulated between foragers and one-day-olds in reference 8 compared to the current study probably reflects a higher statistical power due to increased replication in the former study [8]. Nevertheless, all four species performed comparably on the *A. mellifera *cDNA microarrays and maintained consistent patterns of brain gene expression differences. This demonstrates the utility of the array for carrying out comparisons between *Apis *species.

**Table 1:** Consistency of results compared to an earlier study

		**Genes differentially expressed in Whitfield et al 2006 (p < 0.001)**		
		
**Genes differentially expressed in current experiment (p < 0.01)**		**One-day-old upregulated (1574)**	**Forager upregulated (1678)**	**Replicability**
***A. mellifera***	One-day-old upregulated (665)	413	50	X^2 ^= 562.75 p < 10^-15^
	Forager upregulated (662)	60	432	
***A. cerana***	One-day-old upregulated (425)	302	18	X^2 ^= 400.6 p < 10^-15^
	Forager upregulated (317)	22	214	
***A. dorsata***	One-day-old upregulated (258)	174	18	X^2 ^= 267.53 p < 10^-15^
	Forager upregulated (227)	10	172	
***A. florea***	One-day-old upregulated (528)	326	53	X^2 ^= 402.18 p < 10^-15^
	Forager upregulated (611)	61	356	

Data belonging to each species were analyzed separately to obtain genes significantly regulated between foragers and one-day-olds by an F test (p < 0.01), numbers in parentheses. The comparison set was obtained from Whitfield *et al *2006 [8] and consisted of genes significantly regulated between foragers and one-day-olds (p < 0.001). Only those genes that were present in the gene list of at least one species in the current study were included in the comparison set. Intersects of pair-wise comparisons between the two datasets are given along with results of Chi-square tests.

### Species differences in brain gene expression

1772 genes showed significant differences in brain gene expression between foragers and one-day-olds (ANOVA, F-test, p < 0.001) in any species. However, only 218 genes showed significant difference in forager/one-day-old ratios of brain gene expression (F/DO) between at least 2 species (p < 0.001). At this P value level the number expected by chance alone would be 5.

A pair-wise comparison between species showed that the comparison between *A. florea *and *A. mellifera *resulted in the greatest number of genes (114) showing F/DO differences between any pair of species (Table 2). This is perhaps a reflection of the fact that of the 4 species compared, *A. florea *and *A. mellifera *show the most extreme differences in behavior and ecology (Figure 1). *A. florea *and *A. mellifera *also are among the most distantly related pairs of species in *Apis *[14]. However, the comparison between *A. cerana *and *A. dorsata *resulted in the fewest (18) number of genes showing F/DO differences between any pair of species (Table 2) and these two species also differ extensively in behavior and ecology. However, both *cerana *and *dorsata *are Asian species while *mellifera *is native to Africa and Europe [22].

**Table 2:** Genes significantly regulated between all four species

**Species**	***A. mellifera***	***A. cerana***	***A. florea***	***A. dorsata***
***A. mellifera***	-	37	114	49
***A. cerana***	-	-	51	18
***A. florea***	-	-	-	52
***A. dorsata***	-	-	-	-

Rows above diagonal indicate number of genes with significantly different expression profiles (p < 0.001, F test). 218 genes showed significant differences in expression between at least two species.

**Figure 2:**
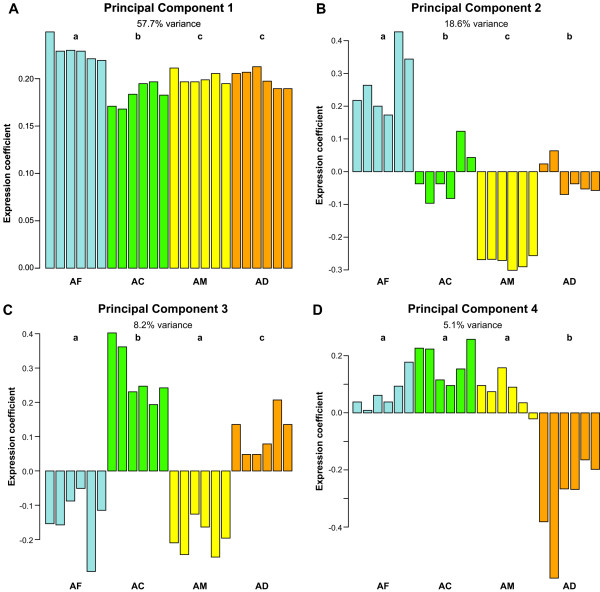
4(A to D): Results of a Principal Component Analysis of the 218 genes expressed significantly differently between at least two species (p < 0.001). Each bar represents a replicate F/DO ratio. Color key/abbreviation: Blue = *Apis florea*/AF, Green = *A. cerana*/AC, Yellow = *A. mellifera*/AM, Red = *A. dorsata*/AD. The order of species is the same in every graph. Different letters of the alphabet depict significantly different mean values according to a post-hoc Tukey's test on an ANOVA of the principal components. Gene loadings on principal components provided in Additional file 1.

A Principal Component Analysis on the brain expression profiles of all 218 genes that showed species differences at p < 0.001 revealed that there were clear patterns of gene expression that differentiated the 4 species from each other (Figure 2). Principal Component 1 (PC1), which accounts for 57.7% of the variance, describes the predominant brain expression pattern as follows: genes that were upregulated or downregulated in forager brains compared to one-day-olds in one species were also likely to be upregulated or downregulated in forager brains compared to one-day-olds in other species (Figure 2a). The major inference that can be drawn from this result is that the molecular processes underlying the maturation of a one-day-old bee to a forager are largely conserved between species. This is consistent with observations suggesting that workers in other *Apis *species show the same basic pattern of behavioral maturation that *A. mellifera *does [16].

PCs 2 and 3, which together accounted for 26.8% of the variance, revealed species differences in brain gene expression that were strong enough to distinguish all the species (Figure 2b and 2c). However, these differences do not overlap with known ecological or physiological differences. For example, the differences do not reflect the fact that two of the species (*florea *and *dorsata) *are open nesting while the other two are cavity nesting; or that all four species differ in size with *dorsata *almost 2.5 fold larger than *florea *[13].

PC 4 (Figure 2d) reflected a pattern in the data that indicates that a component of the variance in brain gene expression is attributable solely to differences between *A. dorsata *and the other species. There are indeed distinctive features of *dorsata *biology relative to the other three species, including migratory habits, defensive behavior, and its much larger size [13], but it is not known whether the differences we detected are related to these aspects.

### Functional classification of differentially expressed genes

To derive inferences (albeit highly speculative) related to functional differences between the species that could guide future studies, we analyzed the 218 genes for overrepresentation in functional Gene Ontology [23] categories (GO, 588 Biological Process terms and 322 Molecular Function terms spanning all levels). Available *Drosophila *orthologs for 145 of the 218 genes were subjected to an enrichment analysis [24] (Table 3).

**Table 3:** Results of an enrichment analysis on 145 genes that show significant differences in expression between the four different species of *Apis*

**GO ID**	**Term**	**Level**	**No. in experiment**	**Expected frequency**	**No. significantly regulated**	**Observed frequency**	**p value**	**Fly orthologs in 147 genes**
GO:0009607	response to biotic stimulus	4	93	0.0513	20	0.1869	1.15E-07	Tctp, Hsp70Aa, e, CG7966, Hsc70Cb, CG32687, Hop, Tsp96F, Hsp83, CG10178, CG5001, Ugt86Dd, Hsc70-4, PGRP-SC2, Gp93, dsd, Tsf1, PebIII, 18w, l(2)efl
GO:0006952	defense response	5	89	0.0491	19	0.1776	2.79E-07	Tctp, Hsp70Aa, e, CG7966, Hsc70Cb, CG32687, Hop, Tsp96F, Hsp83, CG10178, CG5001, Ugt86Dd, Hsc70-4, PGRP-SC2, Gp93, dsd, Tsf1, 18w, l(2)efl
GO:0009408	response to heat	6, 5	12	0.0066	5	0.0467	0.0003452	Hsp70Aa, Hsp83, CG5001, Hsc70-4, l(2)efl
GO:0006457	protein folding	7	45	0.0248	9	0.0841	0.0007291	Hsp70Aa, Hsc70Cb, Hop, Hsp83, FKBP59, CG5001, Hsc70-4, Gp93, l(2)efl
GO:0006950	response to stress	4	65	0.0359	11	0.1028	0.0008152	Hsp70Aa, Hsc70Cb, Hop, Tsp96F, Hsp83, CG5001, Hsc70-4, Gp93, PebIII, 18w, l(2)efl
GO:0042417	dopamine metabolism	9, 8, 6	2	0.0011	2	0.0187	0.0034525	e, Dat
GO:0043473	pigmentation	2	8	0.0044	3	0.028	0.0083358	e, b, Dat
GO:0048066	pigmentation during development	3	8	0.0044	3	0.028	0.0083358	e, b, Dat
GO:0005975	carbohydrate metabolism	5	113	0.0623	12	0.1121	0.0172185	Pglym78, CG6439, CG10178, Mdh, l(2)01810, Sulf1, Ugt86Dd, CG1271, PGRP-SC2, Sodh-2, CG14621, α-Man-IIb
GO:0042752	regulation of circadian rhythm	4, 5	4	0.0022	2	0.0187	0.0183819	Hsp83, Dat
GO:0046916	transition metal ion homeostasis	8	4	0.0022	2	0.0187	0.0183819	Tsf1, CG4349
GO:0006584	catecholamine metabolism	7, 8	4	0.0022	2	0.0187	0.0183819	e, Dat
GO:0006519	amino acid and derivative metabolism	5	67	0.037	8	0.0748	0.0252803	e, CG1732, b, CG6028, CG6439, Eaat2, CG8745, Dat
GO:0009628	response to abiotic stimulus	4	80	0.0441	9	0.0841	0.0254136	Hsp70Aa, wun, Rh6, Hsp83, CG10178, CG5001, Ugt86Dd, Hsc70-4, l(2)efl
GO:0007530	sex determination	3	12	0.0066	3	0.028	0.0258228	fru, gro, CG3726
GO:0009613	response to pest, pathogen or parasite	6, 5	12	0.0066	3	0.028	0.0258228	Tsp96F, PebIII, 18w
GO:0018958	phenol metabolism	6	5	0.0028	2	0.0187	0.0288583	e, Dat
GO:0006807	nitrogen compound metabolism	4	83	0.0458	9	0.0841	0.0302461	e, CG1732, b, CG6028, CG6439, pyd3, Eaat2, CG8745, Dat
GO:0016614	oxidoreductase activity, acting on CH-OH group of donors	4	35	0.0197	7	0.066	0.0029256	CG6439, CG10638, Mdh, CG9360, Sodh-2, antdh, CG10962
GO:0008171	O-methyltransferase activity	6	2	0.0011	2	0.0189	0.0035187	CG10527, Pcmt
GO:0005386	carrier activity	3	131	0.0736	15	0.1415	0.0052529	CG32250, CG31547, CG1732, Cralbp, CG9317, Eaat2, l(3)neo18, Tsp5D, CG33310, l(2)01810, CG1271, Psa, Tsf1, PebIII, CG14621
GO:0051082	unfolded protein binding	4	22	0.0124	5	0.0472	0.0066411	Hop, Hsp83, CG5001, Hsc70-4, Gp93
GO:0004263	chymotrypsin activity	7	3	0.0017	2	0.0189	0.0099384	CG32130, CG4998
GO:0015020	Glucuronosyltransferase activity	6	3	0.0017	2	0.0189	0.0099384	CG10178, Ugt86Dd
GO:0008238	exopeptidase activity	5	17	0.0096	4	0.0377	0.0131342	east, CG4678, Ance, Psa
GO:0015290	electrochemical potential-driven transporter activity	4	48	0.027	7	0.066	0.0149443	CG31547, CG1732, CG9317, Eaat2, l(2)01810, Psa, CG14621
GO:0015291	porter activity	5	48	0.027	7	0.066	0.0149443	CG31547, CG1732, CG9317, Eaat2, l(2)01810, Psa, CG14621
GO:0015293	symporter activity	6	18	0.0101	4	0.0377	0.0159093	CG31547, CG1732, Eaat2, l(2)01810
GO:0016831	carboxy-lyase activity	5	10	0.0056	3	0.0283	0.0162543	b, CG6028, Mdh
GO:0008237	metallopeptidase activity	5	38	0.0214	6	0.0566	0.0165483	Nep2, east, CG4678, Ance, Psa, S2P
GO:0008199	ferric iron binding	7	4	0.0022	2	0.0189	0.0187129	Tsf1, CG4349
GO:0016810	hydrolase activity, acting on carbon-nitrogen (but not peptide) bonds	4	19	0.0107	4	0.0377	0.0189845	pyd3, Sirt2, gro, PGRP-SC2
GO:0008509	anion transporter activity	4	31	0.0174	5	0.0472	0.0251189	CG31547, Eaat2, Atet, l(2)01810, CG14621
GO:0015296	anion:cation symporter activity	7, 5	12	0.0067	3	0.0283	0.0264295	CG31547, Eaat2, l(2)01810
GO:0015370	solute:sodium symporter activity	8, 6	12	0.0067	3	0.0283	0.0264295	CG1732, Eaat2, l(2)01810
GO:0019842	vitamin binding	3	5	0.0028	2	0.0189	0.0293608	Cralbp, CG8745
GO:0005283	sodium:amino acid symporter activity	7, 8, 9, 10, 5	5	0.0028	2	0.0189	0.0293608	CG1732, Eaat2
GO:0004295	trypsin activity	7	5	0.0028	2	0.0189	0.0293608	CG32130, CG4998
GO:0016830	carbon-carbon lyase activity	4	13	0.0073	3	0.0283	0.0323556	b, CG6028, Mdh
GO:0008235	metalloexopeptidase activity	6	13	0.0073	3	0.0283	0.0323556	east, CG4678, Psa
GO:0005279	amino acid-polyamine transporter activity	5, 6	14	0.0079	3	0.0283	0.0387781	CG31547, CG1732, Eaat2

Statistical over-representation of GO categories was tested through a hypergeometric test followed by the Benjamini Hochberg FDR correction using the microarray analysis tool GOToolBox. See Table 6 for honey bee gene IDs

**Figure 3:**
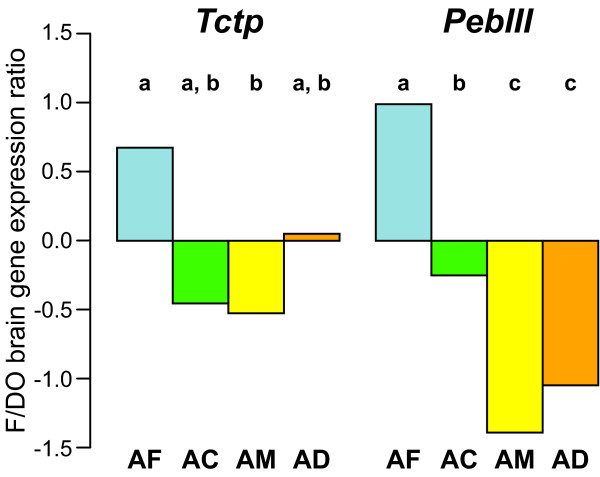
Mean forager by one-day-old brain gene expression ratio (F/DO) of two example fly ortholog genes that show interesting patterns of difference between especially *A. florea *and *A. mellifera*. Species key: AF = *Apis florea*, AC = *A. cerana*, AM = *A. mellifera*, AD = *A. dorsata*. Different letters of the alphabet depict significantly different mean values according to a post-hoc Tukey's test on an ANOVA of the F/DO ratios.

The most significant enrichment among the categories tested was genes implicated in "response to biotic stimulus" and "defense response" (Table 3). This finding is intriguing because these categories encompass responses by a cell or an organism (in terms of movement, secretion, enzyme production, gene expression, etc.) as a result of a stimulus caused or produced by another living organism. Behavioral maturation from one-day-old to forager involves changes in endocrine activity, metabolism, circadian clock activity, brain chemistry and brain structure [25]. In addition, honey bees are highly social organisms and thus highly responsive to their nestmates and conspecifics. One way that honey bees respond to changes in their colony condition is by altering their rate of behavioral maturation [25]; this has only been studied in *A. mellifera*. *Apis *species differ among many other things, including colony size, predator pressure, prevalence of brood diseases and reproductive colony fission [13]. Therefore these groups of highly regulated genes might reflect differences in behavioral maturation processes brought about by differences in social cohesiveness and colony integration. Along these lines, we note that two genes from these two categories, honey bee orthologs of *Drosophila PebIII *and *Tctp*, which have previously been shown to be lower in foragers compared to one-day-olds in *A. mellifera*, [8] showed a reversed and significantly higher F/DO expression ratio in *A. florea *(the open nesting, dwarf species living in small colonies) compared to the other three species (Figure 3).

A couple of other interesting groups of genes were those involved in metabolic processes, e.g., carbohydrate and amino acid metabolism. Similarly, most of the molecular functional categories that emerged as significantly enriched are also groups of genes that participate in metabolic processes, e.g., exo- and metallopeptidases, hydrolases, and genes coding for o-methyltransferases, and carrier proteins (Table 3). This is interesting because one of the most striking differences between the four species relates to differences in worker "tempo." Measurements of colony attributes led Dyer and Seeley [16] to conclude that open-nesting species (*florea *and *dorsata*) have a lower overall level of activity than do the cavity-nesting species (*mellifera *and *cerana*). It is reasonable to speculate that differences in colony activity levels are related to molecular processes associated with worker metabolism. Our results provide potential molecular correlates for these behavioral and ecological observations, and suggest that further analyses of genes in these categories would be particularly fruitful for understanding the ecology of genus *Apis*.

Another category of genes were those implicated in circadian processes. Finding that genes related to circadian rhythms are overrepresented (albeit weakly) on the list of genes showing significant species differences in brain expression is notable from the perspective of honey bee dance language. Brockmann and Robinson [26] discussed possible functional connections between the circadian system and the sun-compass system that is used by honey bees to communicate directional information during dance. The possibility of species differences in these systems is suggested by the fact that *A*. *mellifera*, *cerana *and *dorsata *dance on vertical combs and transpose sun-compass based information to gravity-based information, whereas *A*. *florea *dances on horizontal comb and does not make this transposition.

A more detailed view of the molecular machinery that might underlie species differences in *Apis *was obtained by clustering the 145 orthologous genes that showed significant species differences in brain expression according to their shared functional GO annotation [24]. Several coherent groups of genes emerged from this analysis (Table 4 and 5), in addition to clusters expected due to the enrichment analysis described above (Table 3). Notable among them were the categories of cell communication and development. Genes involved in these processes likely play important roles in brain maturation and sensory development and therefore might contribute to behavioral differences among the species. For example, the honey bee ortholog of *Innexin 3 *(*Inx 3*), a gene whose protein product is important for cell-cell communication [27], is known to be upregulated in young bees when they are treated with the juvenile hormone analog methoprene [8]. Such treatment also induces young bees to become foragers [28]. Our brain transcriptome-wide expression analysis of the four key species of honey bees have provided us with several candidate genes that can be used for much needed comparative studies to uncover the molecular basis of interspecies differences in the genus.

**Table 4:** Functional clustering of 147 fly orthologs of genes showing significant differences in expression between honey bee species, based on Gene Ontology-defined biological processes

**Cluster**	**GO ID**	**GO category**	**Level**	**No. of genes**	**Fly orthologs**	**p value**
1	GO:0007154	cell communication	3	7	Sgt, CG11105, BM-40-SPARC, CG3876, inx3, Cad87A, Mnn1	0.02
2	GO:0015031	protein transport	5,6	5	Kif3C, CLIP-190, garz, CG31048, RhoBTB	5.522e-06
3	GO:0007275	development	2	7	e, Dat, wun, Hsc70-4, bif, Hsp83, chic	0.0148
4	GO:0044237	cellular metabolism	4	17	Tctp, CG10178, Ugt86Dd, Sulf1, CG10903, CG10638, Rpb8, pyd3, ACXD, Gycβ100Bb100B, CG6946, CG7564, Rsf1, fru, CG3726, Sirt2, gro	0.009793
5	GO:0050896	response to stimulus	3	7	CG7966, dsd, Tsp96F, 18w, PebIII, Rh6, CG9265	0.005519
6	GO:0006508	proteolysis	7	5	Nep2, east, CG4998, CG4678, CG32130	0.002551
7	GO:0019538	protein metabolism	5	12	Rpn2, Nep2, east, CG4998, CG4678, CG32130, S2P, Pcmt, RpL6, Cralbp, Psa, FKBP59	2.287e-06
8	GO:0006520	amino acid metabolism	6,7	5	CG1732, Eaat2, CG6028, CG8745, b	0.000116

Clustering was carried out in GOToolBox using the WPGMA algorithm with a Bonferroni correction for multiple testing. A minimum cluster size of 5 genes was applied. See Table 6 for honey bee gene IDs.

**Table 5:** Functional clustering of 147 fly orthologs of genes showing significant differences in expression between honey bee species, based on Gene Ontology-defined molecular functions

**Cluster**	**GO ID**	**GO category**	**Level**	**No. of genes**	**Fly orthologs**	**p value**
1	GO:0008233	peptidase activity	4	6	Rpn2, CG32130, CG4998, CG5798, east, CG4678	3.828e-05
2	GO:0005488	binding	2	15	CG3244, CG7966, CG3104, CG30387, Tsp96F, dsd, Hop, CG5001, CG6946, Rsf1, RpL6, Ef1γ, Rpb8, e, CG8745	0.007796
3	GO:0008092	cytoskeletal protein binding	4	6	Tctp, CLIP-190, coro, CG32030, chic, bif	1.823e-07
4	GO:0005215	transporter activity	2	6	CG32250, Tsp5D, PebIII, CG1358, CG6783, Cralbp	0.007066
5	GO:0008270	zinc ion binding	1	7	CG32486, Pax, Sodh-2, Nep2, S2P, Ance, Psa	1.021e-07
6	GO:0016462	pyrophosphatase activity	6	7	RhoBTB, Hsp83, Hsc70-4, Mnn1, Atet, CG33310, CG1271	1.276e-08
7	GO:0005488	binding	2	21	Tctp, CLIP-190, coro, CG32030, chic, bif, fru, CG3726, gro, CG32486, Pax, Sodh-2, Nep2, S2P, Ance, Psa, Cad87A, CG11105, BM-40-SPARC, Tsf1, CG4349	5.259e-05
8	GO:0003824	catalytic activity	2	33	Rpn2, CG32130, CG4998, CG5798, east, CG4678, wun, CG9265, Sulf1, pyd3, PGRP-SC2, Sirt2, α-Man-IIb, CG11257, CG9360, CG6439, antdh, CG10962, CG10638, b, CG6028, Mdh, ACXD, Gycβ100Bb100B, Pglym78, FKBP59, CG10903, CG10527, Pcmt, CG10178, Ugt86Dd, CG5037, Dat	5.480e-11
9	GO:0016740	transferase activity	3	7	CG10903, CG10527, Pcmt, CG10178, Ugt86Dd, CG5037, Dat	4.211e-06
10	GO:0016787	hydrolase activity	3	13	Rpn2, CG32130, CG4998, CG5798, east, CG4678, wun, CG9265, Sulf1, pyd3, PGRP-SC2, Sirt2, α-Man-IIb	1.016e-07
11	GO:0003676	nucleic acid binding	3	5	CG6946, Rsf1, RpL6, Ef1γ, Rpb8	0.000773
12	GO:0043167	ion binding	3	12	CG32486, Pax, Sodh-2, Nep2, S2P, Ance, Psa, Cad87A, CG11105, BM-40-SPARC, Tsf1, CG4349	1.837e-12
13	GO:0005515	protein binding	3	9	Tctp, CLIP-190, coro, CG32030, chic, bif, fru, CG3726, gro	0.000444
14	GO:0016491	oxidoreductase activity	3	6	CG11257, CG9360, CG6439, antdh, CG10962, CG10638	3.828e-05

See Table 4 legend for details

## Conclusion

This study is the first cross-species comparative study of brain gene expression in honey bees. We used four species of honey bees, three Asian and one European/Western that are known to differ markedly in their nesting habit, behavior and some physiological characters. We compared brain mRNA of foragers and one-day-old bees of each species on each microarray in a replicated loop design.

Performance results for the four species on the microarrays were comparable and in keeping with our current understanding of *Apis *phylogeny [12-14]. A significant fraction of genes in all four species followed expression patterns consistent with a previous study comparing foragers and one-day-olds in *A. mellifera *from Europe [8].

218 genes were found to be expressed significantly differentially between at least two species. Principal Component Analysis revealed strong patterns in the data that grouped the expression data into the four constituent species. Two main inferences could be drawn from the PCA results. First, there appears to be a widespread conservation of the molecular processes in the brain underlying adult honey bee behavioral maturation. Second, the overall pattern of differences did not reflect in an obvious way known differences in behavior and ecology between the four species [13,16]. However, an enrichment analysis for Gene Ontology defined functional categories of genes responsive to biotic stimulus, involved in defense response, metabolism and circadian rhythms–that could plausibly be involved in these ecological differences, as well as in behavioral differences related to dance language.

## Methods

### Honey bee species

The four species of honey bees used for the experiment were all collected from suburban areas of Bangalore, a city in southern peninsular India. Three of these four species are endemic to South Asia, i.e., *Apis cerana *(subspecies *indica*, the South-Asian cavity nesting bee), *A. dorsata *(Asian giant or rock honey bee) and *A. florea *(the Asian dwarf honey bee). The fourth species is *A. mellifera *of Italian descent, introduced in India for commercial beekeeping purposes in the 1960s [29]. All collections of the endemic species were carried out on natural colonies except one colony of *A. cerana *(which was obtained from a commercial beekeeper); *A. mellifera *samples were collected from full-frame hives purchased from a commercial beekeeper.

Returning pollen foragers, easily identified by the brightly colored pollen loads on their hind legs [28], were collected from three colonies of each species mostly during peak foraging hours (10:00–14:00) in the month of November, 2003. After collecting foragers, we transferred pieces of brood comb to an incubator in the lab maintained at 34°C. Freshly eclosed workers were collected over the next 2–3 days within 0–12 h of emergence, referred to as one-day-olds [10]. All samples were collected live and immediately flash frozen in liquid nitrogen. Heads were removed (on dry ice) and stored at -80°C until shipment. Heads were shipped on dry ice to University of Illinois at Urbana-Champaign and stored upon arrival at -80°C until further processing.

### Brain dissections, total RNA extractions and microarray hybridization

Brains were partially lyophilized as in Grozinger et al [30], with the following changes: dissections were carried out in an ethanol bath kept on dry ice and the subesophageal ganglion was retained. Brains were rinsed post-dissection in a fresh bath of ethanol on dry ice to ensure removal of unwanted tissue debris and remnants of hypopharyngeal glands. Pools of 15 brains were homogenized in 1 ml Trizol (Invitrogen) and vortexed with 200 μl chloroform. The mixture was allowed to stand at room temperature for 2 min and then centrifuged at 12,000 g (4°C). The aqueous phase was mixed with 100% ethanol and then transferred to a Qiagen RNeasy column. Subsequent steps for extraction of total RNA were carried out as per kit instructions (Qiagen RNeasy kit for total RNA).

The four species used for the study differ markedly in their brain sizes. Preliminary quantification studies showed that RNA yields from brains differed between the smallest and largest species by as much as 2 times. Therefore, after quantification using a Nanodrop spectrophotometer (ND-1000, Nanodrop Technologies) samples were pooled depending on concentration to obtain uniform yields across species (minimum pool size 30 brains, maximum pool size 60 brains). Total RNA was precipitated in 30 mM sodium acetate and 100% ethanol with 1 μg linear acrylamide, by incubating overnight at -20°C and spinning at 12,000 g for 20 minutes. Pellet was washed in 70% ethanol and air-dried. RNA was resuspended in appropriate volume of RNase-free water and 15 μg was added to 6 μg of dT_18 _primers and annealed at 70°C for 5 min.

Single-strand cDNA was synthesized using 400 U of ArrayScript (Ambion) in an ice cold reaction mixture of 10× 1^st ^Strand buffer, 20 U RNaseOUT (Invitrogen), 0.5 mM dNTP mix (total reaction volume 30 μl), incubated overnight at 42 °C. Reaction was inactivated by incubating at 70°C with 15 μl 0.1 N NaOH for 10 min and neutralized with 15 μl 0.1 N HCl. cDNA was purified using the Qiaquick PCR purification kit (Qiagen) with modified Tris-free buffers [31]and dried down in a SpeedVac. cDNA was resuspended in freshly prepared sodium carbonate buffer and dye coupled as per Whitfield et al 2003 [6], with the exception that dye-coupled samples were pooled only after purification on the Qiaquick PCR purification columns. Four arrays directly compared foragers and day olds of each colony within each species with dye-swap, thus 12 arrays compared brain gene expression of the two behavioral classes for each species (a total of 48 arrays across the experiment, 1 array discarded due to technical problems). Arrays were hybridized and scanned as described in Whitfield et al 2002 [18], arrays were hybridized for 48 h.

### Data analysis

Microarray data generated in this study meet Minimum Information about Microarray Experiment (MIAME) standards and are available at ArrayExpress [32] under accession number E-TABM-262. Data intensity calculations and normalization was carried out as described in Whitfield et al [6], with the exception that the R package MAANOVA was used for carrying out Lowess transformation and the subsequent analysis of variance (ANOVA) [33,34]. All other statistical analyses of the data were also carried out in R. Before normalization, all genes that showed 1.5 times higher expression in hypopharyngeal gland tissue compared to brain tissue were discarded to minimize possible contamination effects as in Whitfield et al 2003 [6]. An initial analysis was performed on each species data separately, applying an identical analysis filter. cDNAs with average expression intensity across all arrays in a species set <300 or absent from >1 array were excluded from the analysis. The species-specific data sets were used to calculate hybridization efficiency and replicability. Finally, in order to enable a combined analysis of all 47 microarrays, a common analysis filter was applied to the data. cDNAs with average expression intensity across all 47 arrays <300 or absent from >1 array were excluded from the analysis. Subsequently, using available genome sequence information [35], ESTs were matched to the official predicted genes from the honey bee genome project using a reverse BLAST procedure and duplicates averaged/collapsed such that each EST represented 1 gene [8]. There were ESTs that had no corresponding match in the predicted gene database and those were retained as such. This resulted in a total of 4432 genes that were used for subsequent analysis. ANOVA was carried out on the log2 transformed intensity values in the two dye channels for all 4432 genes across all arrays. A derived data set was generated using a mixed model with dye and sample (an RNA pool of either foragers or one-day-olds) as fixed factors and array as the random factor [36]. The derived data set was used to calculate ratios of forager to day-old brain expression levels for every gene and each array. A second ANOVA modeling sample (corresponding either to a colony or a species) as a factor was carried out. Finally an F test and a post-hoc Tukey's test were carried out to find gene expression ratios significantly different among the 4 species and between pairs of species, respectively. A Principal Component Analysis (PCA) also was carried out using the singular value decomposition (svd) function in R. The data analyzed were the log2 transformed forager/one-day-old ratios (F/DO) of expression data derived from the two-step ANOVA for the 218 genes significantly regulated between species at p < 0.001. Enrichment of Gene Ontology (GO) [23] categories was statistically tested through a hypergeometric test followed by the Benjamini Hochberg FDR correction using the microarray analysis tool GOToolBox [24]. The input list was 145 known fly orthologs to the 218 genes. The functional clustering of the same genes was also carried out in GOToolBox using the WPGMA algorithm with a Bonferroni correction for multiple testing. A minimum cluster size of 5 genes was applied.

## Authors' contributions

MSS participated in designing the study, executed the experiments, participated in analysis and drafted the manuscript. CWW participated in experimental design and analysis. GER obtained funding for the study, participated in its conception, design and coordination, and helped write the manuscript. All authors have read and approved the final version of this manuscript.

**Table 6:** *Drosophila *orthologs mentioned in Tables 3, 4 and 5 with corresponding honey bee gene IDs

**Fly ortholog (symbol)**	**Fly ortholog (name)**	**Honey bee gene**
Sgt	small glutamine-rich tetratricopeptide containing protein	GB10757-PA
CG11105	CG11105	GB10319-PA
BM-40-SPARC	BM-40-SPARC	BB160012B20F12
CG3876	CG3876	GB14985-PA
inx3	innexin 3	GB10024-PA
Cad87A	Cad87A	GB18254-PA
Mnn 1	Menin 1	GB18223-PA
Kif3C	Kif3C	GB18860-PA
CLIP-190	CLIP-190	GB14183-PA
garz	gartenzwerg	GB12561-PA
CG31048	CG31048	GB19101-PA
RhoBTB	RhoBTB	GB16187-PA
e	ebony	GB19941-PA
Dat	Dopamine N acetyltransferase	GB18080-PA
wun	wun	GB15595-PA
Hsc70-4	Hsc70-4	GB14852-PA
bif	bif CG1822	GB16223-PA
Hsp 83	Heat shock protein 83	GB14758-PA
chic	chico	BB170029A10H06
Tctp	Translationally controlled tumor protein	GB16412-PA
CG10178	CG10178	GB10367-PA
Ugt86Dd	Ugt86Dd	GB17015-PA
Sulf1	Sulfated	GB17701-PA
CG10903	CG10903	GB13406-PA
CG10638	CG10638	GB19030-PA
Rpb8	Rpb8	GB10191-PA
Pyd3	Pyd3	GB20148-PA
ACXD	ACXD	GB20120-PA
Gycβ100B	Guanylyl cyclase β-subunit at 100B	BB170006A10E02
CG6946	CG6946	GB17964-PA
CG7564	CG7564	GB16010-PA
Rsf 1	Repressor splicing factor 1	GB16940-PA
fru	fruitless	GB17617-PA
CG3726	CG3726	GB14649-PA
Sirt2	Sirt2	GB12793-PA
gro	groucho	GB11858-PA
CG7966	CG7966	GB11771-PA
dsd	distracted	GB17759-PA
Tsp96F	Tetraspanin 96F	GB16746-PA
18w	18w	GB16631-PA
PebIII	Ejaculatory bulb protein III	GB18819-PA
Rh6	Rhodopsin 6	GB19657-PA
CG9265	CG9265	GB13292-PA
Nep2	Neprilysin 2	GB16619-PA
east	east CG4399	GB12987-PA
CG4998	CG4998	GB17345-PA
CG4678	CG4678	GB13958-PA
CG32130	CG32130	GB19897-PA
Rpn2	Rpn2	GB10959-PA
S2P	S2P	GB13283-PA
Pcmt	Protein-L-isoaspartate (D-aspartate) O-methyltransferase	GB10967-PA
RpL6	Ribosomal protein L6	GB16628-PA
Cralbp	Cellular retinaldehyde binding protein	GB11326-PA
Psa	Puromycin sensitive aminopeptidase	GB13772-PA
FKBP59	FK506-binding protein FKBP59	GB13770-PA
CG1732	CG1732	BB170016B20A02
like Eaat2	Eaat2	GB16377-PA
CG6028	CG6028	GB12364-PA
CG8745	CG8745	GB13140-PA
b	black	GB19363-PA
CG5798	CG5798	GB15093-PA
CG3244	CG3244	GB14975-PA
CG30387	CG30387	GB12248-PA
Hop	Hsp70/Hsp90 organizing protein homolog	GB19425-PA
CG5001	CG5001	GB18056-PA
Ef1γ	Ef1γ	GB18764-PA
coro	coro	GB20076-PA
CG32030	CG32030	GB13180-PA
CG32250	CG32250	GB13621-PA
Tsp5D	Tetraspanin 5D	BB170022A10E02
CG1358	CG1358	GB18632-PA
CG6783	CG6783	GB15299-PA
CG32486	CG32486	GB19485-PA
CG3104	CG3104	GB19946-PA
Pax	Paxillin	GB19612-PA
Sodh-2	Sodh-2	GB18719-PA
Ance	Angiotensin converting enzyme	GB11983-PA
Psa	Puromycin sensitive aminopeptidase	GB13772-PA
Atet	ABC transporter expressed in trachea	BB170024A10C01
CG33310	CG33310	GB14954-PA
CG1271	CG1271	GB10568-PA
Tsf1	Transferrin 1	GB19745-PA
CG4349	CG4349	GB15667-PA
PGRP-SC2	PGRP-SC2	GB19301-PA
α-Man-IIb	α-Man-IIb	GB10547-PA
CG11257	CG11257	BB160017A10G11
CG9360	CG9360	GB15506-PA
CG6439	CG6439	GB18960-PA
antdh	antdh	GB12522-PA
CG10962	CG10962	GB15662-PA
Mdh	Malate dehydrogenase	GB17291-PA
Pglym78	Phosphoglyceromutase	GB15052-PA
CG10903	CG10903	GB13406-PA
CG10527	CG10527	GB20002-PA
CG5037	CG5037	GB17609-PA
Hsp70Aa	Hsp70Aa	Hsp70Aaxxxxxxx
Hsc70Cb	Hsc70Cb	GB10836-PA
CG32687	CG32687	GB12033-PA
Gp93	Glycoprotein 93	GB12703-PA
l(2)efl	l(2)efl	GB10397-PA
l(2)01810	lethal (2) 01810	GB19693-PA
CG14621	CG14621	GB14064-PA
CG31547	CG31547	GB15653-PA
CG9317	CG9317	GB15192-PA
l(3)neo18	lethal (3) neo18	GB10658-PA

## Supplementary Material

Additional File 1 Supplementary Table 1. Title of Data set – Principal Components Analysis on F/DO brain expression profiles of 218 genes showing significant (p < 0.001) differences in expression between species. Gene loadings for the first 4 Principal Components (PCs), accounting for 89.8% of the variance Click here for file
