# Supplementary material for: Species differences in brain gene expression profiles associated with adult behavioral maturation in honey bees
Source: BMC Genomics. 2007 Jun 29;8:202. doi: 10.1186/1471-2164-8-202 (PMC1929079; doi:10.1186/1471-2164-8-202)
Supplement: Additional File 1 — Supplementary Table 1. Title of Data set – Principal Components Analysis on F/DO brain expression profiles of 218 genes showing significant (p < 0.001) differences in expression between species. Gene loadings for the first 4 Principal Components (PCs), accounting for 89.8% of the variance [file 1471-2164-8-202-S1.doc]

**Supplementary Table 1:** Principal Components Analysis on F/DO brain expression profiles of 218 genes showing significant (p<0.001) differences in expression between species. Gene loadings for the first 4 Principal Components (PCs), accounting for 89.8% of the variance, are indicated.

| **Gene IDs** | **PC1** | **PC2** | **PC3** | **PC4** |
| --- | --- | --- | --- | --- |
| GB10024-PA | -0.05149 | -0.04898 | -0.01256 | 0.069003 |
| GB10038-PA | 0.060767 | -0.14474 | -0.09802 | 0.058072 |
| GB10102-PA | 0.052945 | 0.021408 | -0.0346 | 0.032271 |
| GB10113-PA | -0.03346 | -0.01509 | -0.05481 | -0.09232 |
| GB10191-PA | 0.028889 | 0.022834 | -0.0221 | 0.016477 |
| GB10319-PA | -0.03668 | -0.04341 | 0.041907 | -0.03742 |
| GB10367-PA | 0.086663 | 0.171507 | -0.01088 | 0.121031 |
| GB10397-PA | -0.03332 | -0.17586 | -0.0116 | -0.09228 |
| GB10423-PA | 0.011787 | 0.069346 | -0.05358 | -0.02227 |
| GB10547-PA | -0.13397 | -0.04178 | -0.14591 | 0.059459 |
| GB10568-PA | -0.01064 | -0.01228 | 0.022567 | -0.04702 |
| GB10658-PA | 0.114284 | -0.04481 | -0.09612 | -0.0215 |
| GB10722-PA | 0.077877 | 0.041945 | -0.02568 | -0.02716 |
| GB10757-PA | -0.02084 | -0.06503 | 0.007711 | 0.025903 |
| GB10836-PA | -0.00981 | -0.0713 | 0.019 | -0.07255 |
| GB10959-PA | -0.00902 | -0.06865 | -0.04866 | 0.007001 |
| GB10967-PA | 0.060774 | 0.016439 | -0.05315 | -0.01781 |
| GB10975-PA | 0.075836 | 0.045138 | 0.035805 | -0.07857 |
| GB11111-PA | -0.02892 | -0.05922 | 0.204272 | 0.162083 |
| GB11326-PA | -0.05956 | 0.009867 | 0.035087 | 0.008005 |
| GB11375-PA | -0.14119 | -0.07008 | -0.08806 | -0.05535 |
| GB11496-PA | -0.01396 | 0.068293 | -0.03984 | -0.03265 |
| GB11771-PA | 0.118503 | -0.02717 | 0.06781 | 0.13861 |
| GB11858-PA | 0.026942 | -0.03496 | 0.014197 | -0.04073 |
| GB11983-PA | -0.02831 | 0.054194 | -0.06944 | 0.024817 |
| GB12033-PA | -0.00967 | 0.087034 | -0.02527 | -0.03997 |
| GB12036-PA | 0.014429 | -0.00513 | -0.06178 | -0.02458 |
| GB12248-PA | 0.096734 | -0.00323 | -0.0006 | 0.079443 |
| GB12364-PA | 0.033896 | 0.078726 | -0.05917 | -0.01534 |
| GB12509-PA | -0.03487 | -0.00026 | 0.010723 | -0.05984 |
| GB12517-PA | -0.07091 | 0.028343 | 0.026964 | -0.01099 |
| GB12522-PA | -0.02787 | 0.072603 | -0.09367 | 0.00043 |
| GB12561-PA | 0.047897 | 0.002744 | -0.00595 | -0.04226 |
| GB12703-PA | -0.02914 | -0.12721 | 0.017716 | -0.04488 |
| GB12793-PA | -0.02259 | -0.07409 | 0.038999 | -0.10877 |
| GB12837-PA | -0.01901 | 0.024645 | 0.039976 | -0.04601 |
| GB12987-PA | -0.00491 | -0.01522 | -0.04238 | 0.024198 |
| GB13115-PA | 0.008006 | 0.076499 | -0.03059 | -0.0042 |
| GB13140-PA | 0.115423 | 0.056116 | -0.0049 | 0.123752 |
| GB13157-PA | -0.05753 | 0.040762 | -0.04036 | 0.021495 |
| GB13180-PA | 0.095376 | 0.06805 | 0.070846 | -0.00497 |
| GB13283-PA | -0.02695 | -0.0713 | -0.01252 | 0.054789 |
| GB13292-PA | -0.0326 | 0.103767 | -0.10052 | -0.05077 |
| GB13406-PA | 0.019693 | 0.057923 | -0.04427 | -0.00755 |
| GB13601-PA | -0.20571 | -0.18422 | -0.20804 | -0.28139 |
| GB13621-PA | 0.047836 | 0.100107 | -0.08787 | 0.010065 |
| GB13770-PA | -0.08779 | -0.09884 | 0.026216 | -0.07231 |
| GB13772-PA | 0.013027 | -0.03665 | -0.03822 | 0.02423 |
| GB13852-PA | -0.03745 | -0.08736 | 0.019452 | -0.21886 |
| GB13958-PA | -0.14135 | 0.070671 | -0.10237 | 0.062409 |
| GB14064-PA | 0.142282 | -0.20316 | -0.30126 | 0.037745 |
| GB14166-PA | 0.130938 | -0.05882 | -0.0128 | 0.0866 |
| GB14183-PA | -0.02785 | 0.063824 | -0.03079 | -0.00305 |
| GB14307-PA | -0.01734 | -0.03229 | -0.0121 | 0.047837 |
| GB14470-PA | 0.021358 | -0.06517 | -0.01776 | 0.032131 |
| GB14649-PA | 0.015897 | -0.02128 | 0.062954 | -0.023 |
| GB14740-PA | 0.184394 | 0.028794 | -0.03781 | 0.075767 |
| GB14742-PA | 0.034979 | -0.14133 | 0.025379 | 0.075514 |
| GB14751-PA | -0.07971 | -0.10114 | -0.00823 | 0.074553 |
| GB14758-PA | 0.002505 | -0.10531 | -0.00073 | -0.00257 |
| GB14773-PA | -0.0236 | -0.02532 | 0.010034 | -0.01215 |
| GB14788-PA | 0.017116 | 0.045737 | -0.05647 | 0.057984 |
| GB14852-PA | 0.003973 | -0.08621 | 0.018789 | 0.022961 |
| GB14954-PA | -0.03024 | -0.04145 | -0.02531 | -0.00167 |
| GB14975-PA | -0.0628 | 0.085796 | -0.00776 | -0.07823 |
| GB14985-PA | 0.023287 | 0.066768 | -0.04682 | 0.006421 |
| GB15052-PA | -0.02401 | 0.0757 | -0.03728 | 0.005735 |
| GB15088-PA | 0.035998 | 0.088231 | -0.05828 | -0.00019 |
| GB15093-PA | 0.017749 | -0.06743 | -0.02806 | -0.03297 |
| GB15192-PA | -0.0525 | 0.002329 | -0.11494 | -0.10729 |
| GB15299-PA | -0.04672 | 0.056103 | 0.006218 | 0.033297 |
| GB15429-PA | -0.00792 | -0.01998 | 0.065789 | -0.02336 |
| GB15498-PA | 0.155756 | 0.107241 | 0.061553 | -0.15106 |
| GB15506-PA | 0.073161 | 0.067294 | -0.06668 | 0.046023 |
| GB15529-PA | 0.051207 | 0.037741 | -0.03494 | -0.03251 |
| GB15595-PA | -0.00358 | 0.031638 | 0.017534 | 0.024012 |
| GB15653-PA | 0.067261 | -0.12363 | -0.05712 | -0.05038 |
| GB15662-PA | 0.095033 | -0.07897 | 0.203352 | 0.150667 |
| GB15667-PA | -0.00348 | 0.077704 | -0.03577 | -0.03759 |
| GB16009-PA | -0.06043 | 0.010168 | -0.12958 | -0.03275 |
| GB16010-PA | -0.00948 | 0.017027 | -0.01324 | -0.01193 |
| GB16143-PA | -0.0264 | -0.07824 | -0.0045 | -0.02237 |
| GB16187-PA | 0.011057 | -0.04742 | -0.01051 | 0.002319 |
| GB16223-PA | -0.00152 | -0.07586 | 0.115829 | -0.00988 |
| GB16377-PA | -0.11567 | -0.04775 | -0.0738 | 0.06477 |
| GB16412-PA | -0.00217 | 0.071607 | -0.03646 | -0.05446 |
| GB16541-PA | 0.12955 | 0.009697 | -0.0417 | 0.052795 |
| GB16619-PA | 0.055154 | -0.0038 | -0.30932 | -0.17069 |
| GB16628-PA | 0.019721 | 0.070803 | -0.00587 | -0.06387 |
| GB16631-PA | 0.011548 | -0.06496 | -0.13375 | 0.092338 |
| GB16746-PA | -0.02042 | 0.030037 | -0.01022 | -0.08602 |
| GB16940-PA | 0.008184 | 0.066045 | -0.0509 | -0.02967 |
| GB17015-PA | 0.112734 | 0.035004 | 0.106817 | -0.10193 |
| GB17058-PA | -0.03488 | 0.02442 | -0.12925 | 0.275476 |
| GB17063-PA | 0.052935 | -0.0326 | -0.01191 | 0.089898 |
| GB17140-PA | -0.05472 | -0.07994 | -0.01997 | -0.03111 |
| GB17291-PA | -0.04126 | -0.02946 | -0.0256 | 0.00548 |
| GB17345-PA | -0.04742 | -0.06441 | 0.001683 | -0.04261 |
| GB17390-PA | 0.058409 | 0.026778 | 0.087184 | -0.09032 |
| GB17609-PA | 0.019463 | 0.042312 | -0.02528 | 0.006937 |
| GB17617-PA | 0.015285 | 0.025156 | 0.037848 | -0.02703 |
| GB17701-PA | -0.02342 | 0.087351 | -0.08397 | -0.02116 |
| GB17759-PA | 0.005228 | -0.03146 | -0.02355 | 0.022746 |
| GB17835-PA | 0.0434 | -0.06719 | -0.03638 | 0.059129 |
| GB17964-PA | 0.031838 | 0.006347 | -0.04185 | -0.00488 |
| GB18056-PA | 0.060518 | -0.08818 | 0.024664 | -0.03021 |
| GB18080-PA | 0.003467 | 0.037367 | -0.03318 | -0.04742 |
| GB18167-PA | 0.010968 | 0.052905 | -0.0471 | -0.01339 |
| GB18223-PA | 0.022647 | -0.08185 | -0.06949 | 0.069929 |
| GB18237-PA | -0.06229 | 0.068339 | -0.12372 | -0.04689 |
| GB18254-PA | -0.00774 | -0.03831 | 0.012183 | -0.0399 |
| GB18489-PA | 0.078485 | 0.045801 | 0.01192 | -0.07822 |
| GB18632-PA | -0.08145 | 0.001658 | -0.08185 | 0.050635 |
| GB18719-PA | 0.083105 | 0.076039 | -0.14685 | -0.0523 |
| GB18764-PA | -0.01218 | 0.051298 | -0.02603 | -0.01815 |
| GB18819-PA | -0.03161 | 0.132474 | -0.02623 | 0.072283 |
| GB18860-PA | -0.00484 | -0.038 | -0.01348 | -0.02603 |
| GB18957-PA | -0.01386 | -0.01479 | 0.030608 | -0.03413 |
| GB18960-PA | -0.02459 | 0.049801 | -0.05446 | 0.017797 |
| GB19030-PA | 0.056189 | 0.061855 | -0.05386 | -0.13321 |
| GB19101-PA | -0.02058 | -0.05077 | -0.00635 | 0.048627 |
| GB19301-PA | 0.102636 | -0.01294 | -0.04516 | -0.10109 |
| GB19363-PA | 0.041567 | 0.105404 | -0.05749 | 0.025152 |
| GB19425-PA | -0.00202 | -0.06227 | 0.019011 | -0.04954 |
| GB19485-PA | -0.03133 | -0.04433 | 0.01906 | -0.032 |
| GB19612-PA | -0.00207 | -0.03901 | -0.02884 | 0.006033 |
| GB19644-PA | 0.100502 | 0.055942 | -0.02799 | -0.03313 |
| GB19657-PA | 0.007978 | 0.010829 | 0.178377 | -0.06007 |
| GB19693-PA | -0.02813 | 0.020296 | -0.02745 | -0.14349 |
| GB19745-PA | 0.329695 | 0.021901 | -0.16371 | 0.009367 |
| GB19828-PA | 0.018376 | 0.059658 | -0.09334 | 0.046101 |
| GB19897-PA | 0.008153 | -0.09888 | 0.011435 | -0.08149 |
| GB19941-PA | 0.037357 | 0.065112 | -0.02799 | -0.07469 |
| GB19946-PA | 0.04826 | 0.092998 | -0.02933 | 0.003293 |
| GB20002-PA | 0.03297 | 0.036773 | -0.0619 | 0.04162 |
| GB20076-PA | -0.07014 | 0.022274 | 0.003533 | -0.05319 |
| GB20114-PA | 0.021246 | 0.0555 | -0.03651 | 0.002986 |
| GB20120-PA | 0.035093 | 0.036012 | -0.05862 | 0.072058 |
| GB20148-PA | -0.0006 | 0.101711 | -0.08018 | -0.02231 |
| BB160014B20D09 | 0.178743 | -0.11193 | -0.0704 | -0.0904 |
| BB160021A10A05 | -0.0581 | 0.014874 | -0.03581 | 0.042709 |
| BB170016B20A02 | -0.17485 | 0.041329 | -0.11846 | 0.036366 |
| BB170021B10A02 | -0.03019 | 0.018774 | 0.025404 | -0.09264 |
| BB160015B10C11 | 0.046659 | -0.05952 | -0.05009 | 0.044358 |
| BB170025B10A01 | 0.043953 | -0.02844 | -0.02617 | 0.041926 |
| BB170030A20F01 | 0.048564 | -0.02958 | -0.05868 | 0.053744 |
| BB160023B20H08 | 0.033682 | -0.07509 | -0.00686 | 0.01285 |
| BB160013B10G03 | -0.02942 | -0.0538 | -0.01031 | -0.01738 |
| BB170025B20F02 | -0.13645 | 0.066709 | -0.05522 | -0.0488 |
| BB160006B20D10 | 0.059212 | 0.019302 | -0.02721 | -0.04126 |
| BB160019A20A07 | -0.00949 | -0.05229 | -0.04933 | -0.00022 |
| BB170013B10C06 | 0.047489 | -0.02223 | -0.03747 | 0.053286 |
| BB170004A20A07 | -0.07213 | -0.06098 | 0.018412 | 0.033065 |
| BB170004A20A01 | 0.021026 | -0.01281 | 0.072393 | -0.14991 |
| BB160006A20B11 | -0.00434 | -0.0308 | -0.10535 | 0.045824 |
| BB170027B10C08 | -0.05267 | -0.02844 | -0.07364 | 0.027371 |
| BB170001B10G06 | -0.06855 | -0.10497 | -0.04055 | 0.17418 |
| BB170003A10D08 | -0.02681 | 0.039187 | -0.05898 | 0.03372 |
| BB160020B20D10 | -0.03096 | 0.04073 | -0.00743 | 0.000836 |
| BB170029A10H06 | 0.025023 | -0.05942 | 0.01637 | -0.02421 |
| BB160008B10G06 | 0.023987 | -0.04633 | -0.05133 | 0.047333 |
| BB160020B20F04 | 0.000644 | -0.05049 | -0.01469 | 0.086922 |
| BB170015B20E11 | -0.0088 | 0.023251 | -0.05932 | 0.017814 |
| BB170009A10A09 | 0.021646 | -0.07547 | 0.026358 | -0.05696 |
| BB170013A20G10 | 0.037931 | -0.14412 | -0.00283 | 0.090114 |
| BB170016B20B07 | 0.078754 | 0.067644 | -0.03006 | -0.07169 |
| BB170026A20C04 | 0.021614 | -0.14823 | 0.047559 | 0.058236 |
| Hsp70Aaxxxxxxx | 0.00949 | -0.11219 | 0.023994 | 0.005202 |
| BB170032B20G12 | 0.024832 | 0.025226 | -0.02461 | 0.022577 |
| BB160020A20A11 | 0.068402 | -0.05366 | 0.013908 | -0.00367 |
| BB170022A10E02 | -0.08818 | -0.08419 | -0.04385 | 0.09495 |
| BB160021B20B09 | -0.00826 | 0.07957 | -0.0213 | -0.06359 |
| BB170023A10G10 | -0.03719 | -0.0011 | -0.07817 | 0.000928 |
| BB170024A10C01 | 0.047084 | -0.01112 | -0.0395 | 0.043178 |
| BB170008B20A03 | -0.03469 | 0.013751 | -0.05977 | 0.059382 |
| BB160006A20H11 | 0.206828 | -0.11752 | -0.02904 | -0.12935 |
| BB170017B10C05 | 0.083669 | -0.0652 | 0.0643 | -0.14004 |
| BB160005B20C12 | 0.052748 | -0.05931 | -0.00557 | 0.042481 |
| BB160013A20H08 | -0.00608 | 0.008389 | 0.039828 | -0.03148 |
| BB160017A10G11 | 0.007779 | -0.02139 | -0.05874 | 0.038445 |
| BB170018B20A11 | -0.01646 | -0.06209 | -0.01571 | 0.015127 |
| BB170025B20F05 | 0.001478 | -0.05337 | 0.057353 | -0.02788 |
| BB160019A20H06 | 0.010029 | 0.04099 | -0.02945 | -0.00342 |
| BB170006A10E02 | -0.03127 | -0.06335 | -0.06072 | 0.047661 |
| BB170021A20E08 | -0.02166 | -0.00069 | 0.046256 | -0.07028 |
| BB170010A10F11 | 0.075063 | -0.15377 | -0.09003 | 0.04237 |
| BB170028B20D03 | -0.0105 | -0.02382 | 0.003044 | -0.00382 |
| BB160008A10E09 | 0.013318 | -0.07419 | 0.095873 | -0.0676 |
| BB160012B20F12 | -0.22735 | -0.02375 | -0.10335 | 0.122164 |
| BB170027A10B02 | -0.03558 | -0.14901 | -0.01816 | -0.0913 |
| BB160021A20A07 | -0.02361 | -0.03834 | -0.04701 | 0.03394 |
| BB170005B10H04 | -0.0205 | -0.02842 | -0.02832 | 0.041422 |
| BB170010B10G12 | 0.100909 | -0.09334 | -0.05993 | -0.00225 |
| BB170023B20G09 | 0.027831 | 0.1267 | 0.048417 | 0.025875 |
| BB170004B10H06 | -0.03322 | -0.01458 | 0.045028 | -0.05826 |
| BB170027B10F08 | 0.042499 | -0.00246 | 0.156782 | 0.15332 |
| BB170029B20D05 | -0.0939 | -0.00376 | 0.020026 | 0.0334 |
| BB160007B20E07 | -0.03295 | 0.016465 | -0.07 | 0.019153 |
| BB160014B20E11 | 0.124027 | 0.005272 | -0.02557 | 0.057041 |
| BB160019B10F10 | -0.00491 | 0.080776 | -0.04585 | 0.011889 |
| BB160012B10C04 | 0.000303 | -0.03412 | 0.000134 | -0.0112 |
| BB170028A10E08 | -0.00762 | 0.033489 | -0.01908 | -0.02844 |
| BB160006B10G12 | 0.008338 | -0.02228 | -0.0946 | 0.036016 |
| BB170013B10D01 | 0.123244 | 0.00854 | -0.03683 | 0.053332 |
| BB170013A20D07 | 0.005186 | -0.08028 | 0.038733 | 0.078215 |
| BB170026A20F12 | -0.02928 | 0.02504 | -0.0358 | -0.01297 |
| BB160006A20E01 | 0.022701 | -0.05788 | -0.03069 | 0.028178 |
| BB160010A20E06 | -0.01265 | -0.05101 | -0.02224 | 0.047627 |
| BB170029B10E07 | -0.02328 | 0.045016 | -0.03628 | 0.011546 |
| BB170031B20E05 | 0.0405 | -0.12922 | -0.05168 | 0.014783 |
| BB170013B10A05 | -0.09433 | -0.05268 | -0.06923 | -0.04503 |
| BB170012B20B02 | -0.06084 | -0.04267 | 0.108926 | -0.0338 |
| BB160015A20H11 | 0.005572 | -0.10505 | 0.012215 | -0.0629 |
| BB160024B20C06 | 0.02753 | -0.04593 | -0.03455 | 0.049673 |
| BB170014B10E02 | 0.014488 | 0.02885 | -0.0168 | 0.061875 |
| BB170025A10C08 | 0.021367 | -0.0453 | -0.05903 | 0.071634 |
| BB160017B20H07 | 0.210106 | -0.11819 | -0.05698 | -0.11121 |
| BB170022B20B01 | 0.079655 | -0.03498 | -0.08305 | -0.00787 |
